# The Novel Soluble Guanylate Cyclase Stimulator Attenuates Acute Lung Injury via Inhibiting Pericyte Phenotypic Transition

**DOI:** 10.3390/ijms27031346

**Published:** 2026-01-29

**Authors:** Yu-Long Huang, Shuo Li, Xia Li, Jin-Shui Zhang, Ying-Xian Shi, Gui-Xin Su, Yang Zhang, Rui Xue, Jing-Cao Li, Qiong-Yin Fan, Zhi-Bing Zheng, Yun Deng, You-Zhi Zhang

**Affiliations:** 1 Beijing Institute of Pharmacology and Toxicology, Beijing 100850, China; 18176702599@163.com (Y.-L.H.); shuoli1204@163.com (S.L.); lixia199156@163.com (X.L.); zhangjinshui991023@163.com (J.-S.Z.); syx15138911232@163.com (Y.-X.S.); sgx07012024@163.com (G.-X.S.); zhangyang@bmi.ac.cn (Y.Z.); hly19830718@sina.com (R.X.); lijing-cao00@126.com (J.-C.L.); bingofqy@163.com (Q.-Y.F.); zbzheng@nic.bmi.ac.cn (Z.-B.Z.); 2 School of Medicine, Anhui University of Science and Technology, Huainan 232001, China

**Keywords:** acute lung injury, riociguat, sGC003, sGC, pericyte, phenotype transition

## Abstract

Acute lung injury (ALI) pathogenesis is intricately linked to microvascular permeability. Soluble guanylate cyclase (sGC) is prominently expressed in the vascular system, playing a central role in vascular function. In contrast, its expression and function diminish notably during the progression of ALI, indicating sGC’s potential significance as a pivotal modulator in the pathological processes of ALI. Nonetheless, the precise localization of sGC within lung tissue and its distinct mechanism in maintaining vascular homeostasis remain unclear. Furthermore, there is a necessity for a pharmacological agent capable of consistently activating sGC for the treatment of ALI. A novel sGC stimulator, sGC003, was engineered through structural modification of Riociguat. In a mouse model of ALI, sGC003 exhibited superior sGC activation and more potent anti-inflammatory effects relative to Riociguat. It also exhibited superior efficacy in improving respiratory function and reducing pulmonary edema. Through single-cell RNA sequencing and immunofluorescence co-localization analysis, we confirmed predominant expression of soluble guanylate cyclase in pericytes. The sGC stimulators were found to modulate the LPS-induced pericyte transcriptome reprogramming via the nitric oxide (NO)-sGC-cyclic guanosine monophosphate (cGMP) pathway. Differential gene expression analysis categorized pericytes into nine distinct subgroups, which were sequentially activated during vascular development, inflammation, and myofibrosis. Pseudotime analysis revealed that sGC003 more effectively suppressed the myofibroblast differentiation of pericytes compared to Riociguat. In conclusion, sGC003 mitigates ALI-induced pulmonary inflammation by modulating pericyte differentiation, particularly in preserving microvascular integrity outstanding performance. Its exceptional efficacy suggests that it could potentially serve as a safer and more efficient option as a novel sGC stimulant in the future.

## 1. Introduction

Acute pulmonary edema represents a common complication of acute lung injury (ALI) and serves as a primary cause of acute respiratory distress syndrome (ARDS) [1,2]. ALI damages the pulmonary vascular barrier, leading to edema in the alveolar and interstitial spaces [3]. ALI is driven by several pathophysiological mechanisms, including inflammatory mediator release, alveolar epithelial cell and endothelial cell (EC) dysfunction, and changes in pericyte and microvascular EC interactions [4,5,6]. Under normal conditions, the pulmonary vascular barrier is maintained by ECs, the basement membrane, and pericytes, which ensure effective gas exchange and selective permeability [7,8]. During the development of ALI/ARDS, inflammation or direct injury increases vascular barrier permeability [9], allowing plasma components and inflammatory cells to leak into the alveoli and interstitial spaces, worsening pulmonary edema, and impairing gas exchange. ALI and ARDS are associated with pneumonia and sepsis, and severe ARDS is marked by a substantial case-fatality burden, with rates estimated at around 40% [10,11,12]. The effectiveness of current treatments in alleviating the severity of acute pulmonary edema and reducing mortality is limited. Furthermore, extensive use of corticosteroids often causes significant side effects [13].

Pulmonary edema is clinically characterized by rapid progression of lung injury, indicating that the initial alveolar–capillary barrier damage accelerates the pathological process [14]. Lipopolysaccharide (LPS) and hypoxia induce pericyte cytoskeletal rearrangement, resulting in reduced pericyte coverage of ECs and impaired EC–pericyte interactions. These changes in pericyte–endothelial interactions lead to increased vascular leakage and heightened inflammatory responses [15]. In ALI mouse models, differentially expressed genes linked to leukocyte trafficking and cytokine production are significantly enriched in pericytes, highlighting the critical role of pericytes in regulating local inflammatory responses [6]. Stasi et al. demonstrated that alpha smooth muscle actin (α-SMA)^+^ immunostaining is present in vascular smooth muscle cells of healthy pigs, but not in pericytes; however, α-SMA^+^ cells increase significantly approximately 24 h after the onset of inflammatory infiltration in pigs with endotoxemia [16]. Moreover, studies have demonstrated that α-SMA serves as a marker of myofibroblasts [17]. In a study of the transdifferentiation of pericytes and myofibroblasts within the kidney, Wu et al. showed that inactivation of signaling pathways between ECs and pericytes induces pericyte phenotypic changes and disease progression [18]. In an LPS-induced ALI mouse model, vascular integrity is compromised, while transforming growth factor (TGF)-β1 overexpression causes pericyte phenotype transformation from platelet-derived growth factor receptor (PDGFR)-β to α-SMA, alongside substantial collagen deposition [19,20,21]. Thus, impaired pericyte function caused by pericyte-myofibroblast transition may lead to their separation from microvascular ECs and reduced coverage, exacerbating ALI. Dysfunctional pericytes are involved in sepsis-induced microvascular dysfunction and vascular leakage, key indicators of end-organ dysfunction and septic shock [22]. Although novel strategies targeting metabolism, stem cell therapy, and nanodelivery have emerged in the field of ALI treatment and demonstrated potential in preclinical studies, these approaches commonly face shared challenges in targeting specificity, safety, and translational feasibility [23,24,25]. Consequently, exploring therapeutic targets with well-defined mechanisms and clear intervention pathways is of considerable importance. In recent years, research focus has gradually shifted toward the regulation of vascular barrier stability, where the interaction between endothelial cells and pericytes has been recognized as a critical element in maintaining microvascular integrity [26,27]. Experimental evidence indicates that pericyte detachment in the early stages of ARDS is directly associated with barrier disruption, and supporting their adhesion and communication through specific signals can effectively mitigate pulmonary edema [22]. Despite the established role of EC–pericyte interactions in vascular homeostasis, systematic research aimed at translating this knowledge into reliable therapeutic approaches remains scarce, and much of the literature on clinical ALI treatment remains centered on anti-inflammatory strategies [28]. Therefore, this study focuses on developing ALI therapies through the stabilization of EC–pericyte interactions, aiming to provide a novel clinical pathway for ALI treatment based on vascular barrier stabilization.

In an LPS-induced ALI mouse model, the resulting dysfunction in nitric oxide (NO) and soluble guanylate cyclase (sGC) signaling leads to loss of endothelial integrity, leakage, inflammatory cell infiltration into the surrounding tissues, severe pulmonary edema, and hemorrhagic spots [6,29,30,31]. Riociguat is an sGC stimulator developed by Bayer Healthcare Ag (Berlin, Germany) to treat pulmonary hypertension [32]. Treatment with riociguat restores NO-sGC signaling and significantly attenuates the development of edema and hemorrhagic spots in the LPS-induced ALI mouse model [6]. Similar effects have also been observed with other sGC activators [33,34,35,36,37]. Therefore, sGC activation prevents inflammation-induced pericyte cytoskeletal rearrangement and pericyte detachment from alveolar capillaries, resulting in maintenance of vascular barrier function.

A novel sGC stimulator, methyl 4,6-diamino-2-[1-[(3-fluorothiophen-2-yl)methyl]-1H-pyrazolo [3,4-b]pyridin-3-yl]-5-(methylamino)pyrimidine-5-carboxylate (sGC003), was developed by modifying the structure of riociguat. Initially, the pyrazolo [3,4-b]pyridine core was constructed via cyclization of p-methoxybenzylhydrazine with ethyl cyanopyruvate sodium salt, followed by cerium(IV) ammonium nitrate (CAN)-mediated deprotection to afford the key intermediate 3-amino-1H-pyrazolo[3,4-b]pyridine. Concurrently, the 2-chloromethyl-3-fluorothiophene side chain was prepared from methyl 2-aminothiophene-3-carboxylate through a Schiemann reaction, reduction, and chlorination. Following N-alkylation to connect both fragments, the intermediate was converted to an amidine via a Pinner reaction, which then underwent cyclization with phenylazomalononitrile to form the pyrimidine ring. Catalytic hydrogenolytic deprotection yielded a triamine hydrochloride intermediate. Finally, successive reactions with methyl chloroformate (carbamoylation) and iodomethane in the presence of lithium hexamethyldisilazide (LiHMDS) for N-methylation, furnished the target compound, sGC003. All key intermediates and the final product were characterized and confirmed by ^1^H-NMR and mass spectrometry, with further details available in patent CN102485724A [38]. The objective of this study was to determine the therapeutic effects of sGC003 on ALI, prior to which we have preliminarily found that the drug has certain therapeutic and preventive effects on pulmonary edema caused by high-altitude environment and has applied for patent authorization (CN118217290A). We established a mouse ALI model to investigate the in vivo effects of sGC003. Single-cell RNA sequencing was employed to precisely identify relevant target cells, thereby elucidating the mechanisms underlying ALI and evaluating the efficacy of sGC003. This study suggests that sGC003, a novel sGC stimulator, may offer a safer, more effective preventive approach for lung-injury-related diseases, with potentially fewer adverse reactions.

## 2. Results

### 2.1. Molecular Dynamics Simulations Analysis

**Figure 1:**
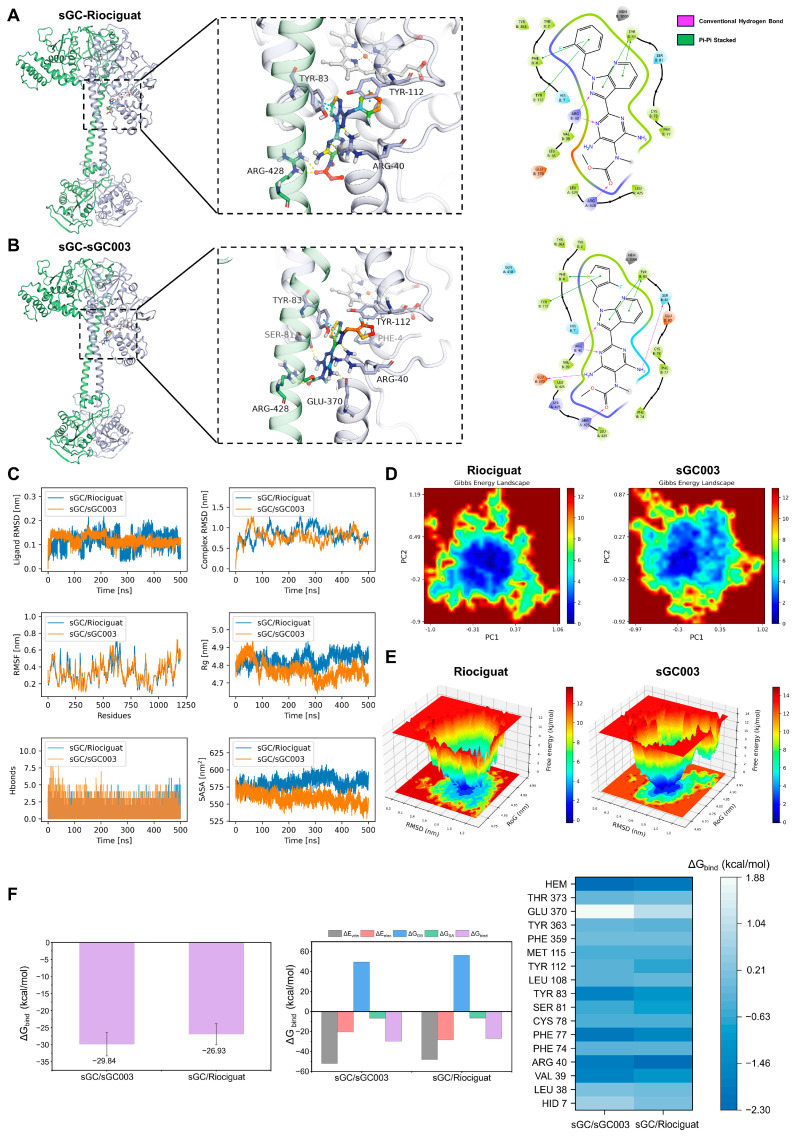
Molecular dynamics simulations. (**A**) Protein–ligand interactions of riociguat and sGC. (**B**) Protein–ligand interactions of sGC003 and sGC. (**C**) Structural stability analysis. (**D**) Gibbs energy landscape analysis. (**E**) FEL analysis. (**F**) Binding free energy calculation. In (**A**,**B**), the left images show overall views, and the right images show detailed views. Green sticks represent small molecules; yellow dashed lines indicate hydrogen bonds; cyan dashed lines depict pi–pi stacking interactions.

Molecular docking studies revealed that riociguat forms three hydrogen bonds with ARG-428 and ARG-40, and engages in pi–pi stacking interactions with TYR-112 and TYR-83 of the sGC protein (Figure 1A). sGC003 forms five hydrogen bonds with SER-81, ARG-40, ARG-428, and GLU-370, and participates in pi–pi stacking with TYR-112, TYR-83, and PHE-4 (Figure 1B). These results indicate that both riociguat and sGC003 bind to sGC, with sGC003 exhibiting stronger binding affinity. To further characterize these interactions, we performed molecular dynamics (MD) simulations to assess the conformational stability and temporal dynamics of the resulting ligand–protein complexes. To ensure the reliability of the energy analysis, the convergence of the MD simulations was carefully evaluated. After equilibration, the observed fluctuations in RMSD and RMSF primarily reflect the intrinsic conformational dynamics of the biomolecules, rather than global system instability. This indicates that the MMGBSA results derived from the equilibrated trajectory are reproducible and physically meaningful. As Figure 1C shows, the ligand root mean square deviation (RMSD) of sGC003 remained consistently lower with less fluctuation compared to riociguat, indicating more stable binding of sGC003 to the sGC protein, while riociguat exhibited noticeable conformational drift. The lower RMSD values suggest that sGC003 maintained a relatively fixed position within the binding pocket, implying stronger interactions with the protein. In contrast, riociguat showed greater flexibility in its binding mode, though with reduced stability. The complex RMSD values indicated that both the sGC003–sGC and riociguat–sGC complexes stabilized after 300 ns of simulation, with minor differences, suggesting that both complexes can maintain overall stability. Root mean square fluctuation (RMSF) values provided insight into the flexibility of individual residues within the ligand–protein complexes. The fluctuation profiles of protein residues were similar in both systems, with most RMSF values below 0.5 nm, indicating low protein flexibility that facilitates stable binding of both small molecules. The radius of gyration (Rg) values reflected the compactness of the ligand–protein complexes. The sGC003 complex exhibited slightly lower and less variable Rg values than the riociguat complex, suggesting that sGC003 helps maintain structural compactness and reduces the tendency for conformational loosening. This implies that sGC003 binding supports the overall tertiary fold of the protein and decreases the potential for unfolding during extended simulations. In comparison, the riociguat complex showed somewhat larger structural fluctuations, reflecting its lower capacity to stabilize the complex conformation. Solvent accessible surface area (SASA) values indicated the solvent exposure of the protein–ligand complexes. The sGC003–sGC complex had generally lower and less fluctuating SASA values than the riociguat complex, indicating reduced solvent-accessible surface area and supporting a tighter and more stable binding mode. This suggests that the sGC003–sGC complex buries more hydrophobic core regions, reducing non-specific interactions with the solvent. In contrast, the higher SASA of the riociguat complex implies greater solvent exposure of its binding pocket and lower stability relative to the sGC003 complex.

Additionally, the sGC003–sGC system formed a greater number of hydrogen bonds in the later stages of the simulation, which may partly explain its superior binding compared to riociguat. To understand the potential binding states, flexibility, and energy distribution of the ligands within the protein active site, we constructed free energy landscape (FEL) based on PCA and RMSD (Figure 1D,E). In the sGC–riociguat system, the low-free-energy regions (blue areas) were relatively dispersed with less concentrated energy basins. In contrast, the sGC–sGC003 system showed a deeper and more concentrated energy basin with tightly distributed low-energy regions. These findings are consistent with the RMSD, Rg, and hydrogen bond results, further supporting the conclusion that sGC003 binds more stably to sGC. Additionally, binding free energies were computed from the MD trajectories using the MM-GBSA method. As Figure 1F shows, the binding energy of sGC–sGC003 was lower than that of sGC–riociguat. Energy decomposition showed that van der Waals interactions emerged as the main contributor to binding in both complexes, with electrostatic interactions and nonpolar solvation free energy following in contribution. Per-residue energy decomposition analysis identified residues contributing critically to binding within the active site. sGC003 showed substantial energy contributions from residues such as HEM and PHE 77, while riociguat exhibited a strong contribution from ARG 40; other residues mainly provided less extensive energy contributions, maintaining stable binding profiles. These results indicate that sGC003 possesses superior binding affinity compared to riociguat.

### 2.2. Effects of sGC003 on the Phenotype of LPS-Induced ALI in Mice

**Figure 2:**
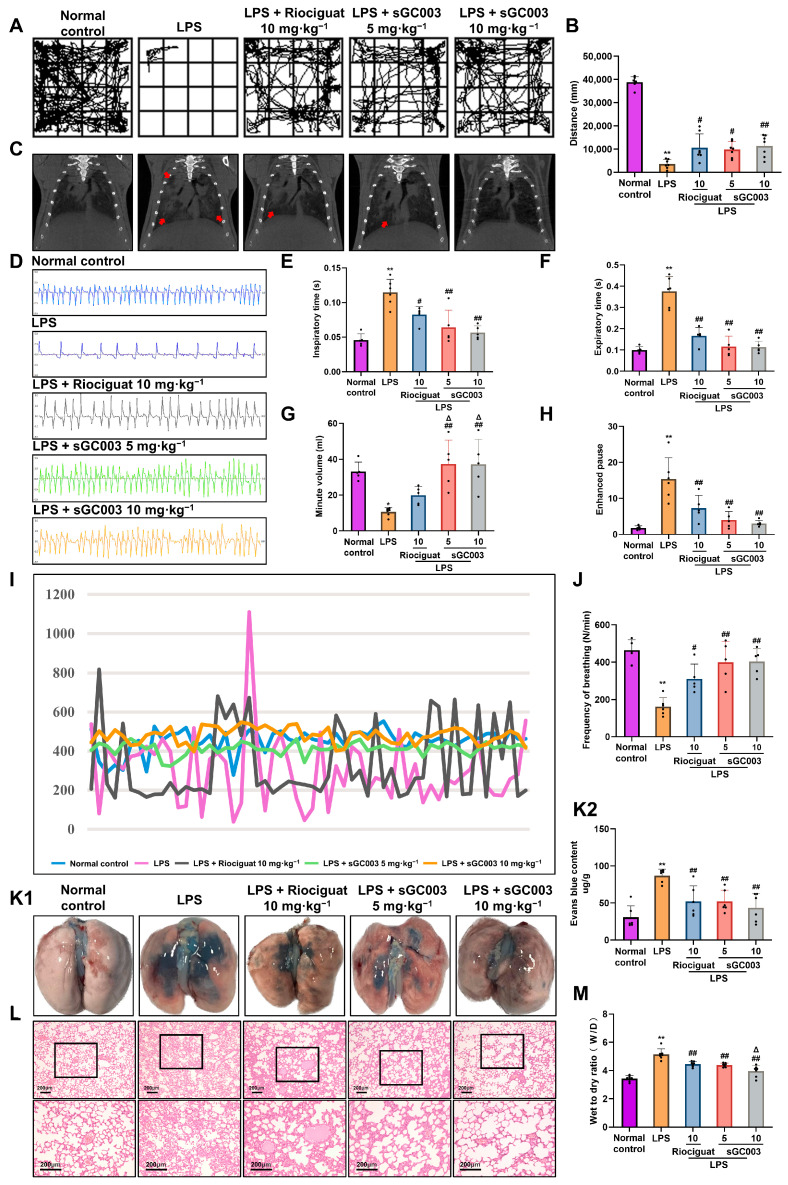
SolubleGC003 protects against LPS-induced acute lung injury (ALI) in mice. (**A**) The movement of mice was detected using an open-field test. (**B**) The total distance traveled (mm), *n* = 7. (**C**) Micro-computed tomography scan results. (**D**) Mouse respiratory waveforms. (**E**) Inspiratory time, *n* = 5–6. (**F**) Expiratory time, *n* = 5–6. (**G**) Minute volume, *n* = 5–6. (**H**) Enhanced pause, *n* = 5–6. (**I**,**J**) Frequency of breathing, *n* = 5–6. (**K1**) Evans blue staining of lungs. (**K2**) Quantification of (**K1**), *n* = 6. (**L**) H&E staining, scale bar = 200 µm. (**M**) Wet/dry weight ratios of the lungs, *n* = 7. The red arrows in (**C**) represent the colocalization of lung injury. Black box indicates the enlarged view of a representative area. Data are presented as means ± SD. # *p* < 0.05, ## *p* < 0.01, vs. LPS group; * *p* < 0.05, ** *p* < 0.01, vs. Normal control group; Δ *p* < 0.05 vs. Riociguat group.

Movement distance in the ALI group was significantly shorter than movement distance in the normal control group, and treatment with sGC003 and riociguat attenuated these effects (Figure 2A,B). The micro-CT imaging revealed diffuse infiltration and pronounced enhancement in the lungs in the ALI model, indicating the occurrence of ALI and edema. Treatment with riociguat and sGC003 significantly reduced lung density and the extent of diffuse infiltrative shadows (Figure 2C). Whole-body plethysmography showed that the duration of apnea and the depth of breathing increased in the ALI model. Administration of riociguat and sGC003 attenuated these effects (Figure 2D). Due to the severe decline in lung compliance, mice exhibited slower and deeper breathing to enhance respiratory efficiency. Inspiratory time (Ti) and expiratory time (Te) were significantly prolonged, minute volume (Mv) decreased, and enhanced pause (Penh) increased in the LPS-treated mice compared to the normal control group. Treatment with sGC003 alleviated the enhanced pause (Penh), increased Mv, and shortened the durations of Ti and Te compared with the LPS-treated mice (Figure 2E–H). Frequency of breathing was significantly reduced and occasional episodes of brief tachypnea and worsened respiratory stability occurred in the ALI mice. Treatment with sGC003 restored normal frequency of breathing (F/N) (Figure 2I,J). Extensive Evans blue staining was observed in the LPS group, indicative of severe vascular leakage. Both sGC003 and riociguat significantly reduced the blue staining compared with the ALI model group (Figure 2K1,K2). Lung wet-to-dry weight ratios, indicating pulmonary edema, were reduced after treatment with riociguat and sGC003 compared with the LPS group; sGC003 exhibited more pronounced effects than riociguat (Figure 2M). H&E staining revealed that riociguat and sGC003 reduced alveolar exudation, maintained alveolar homeostasis, and mitigated the damage caused by ALI and acute pulmonary edema (Figure 2L).

### 2.3. Single-Cell RNA Sequencing Analysis and Annotation of Lung Tissues from ALI Mice

**Figure 3:**
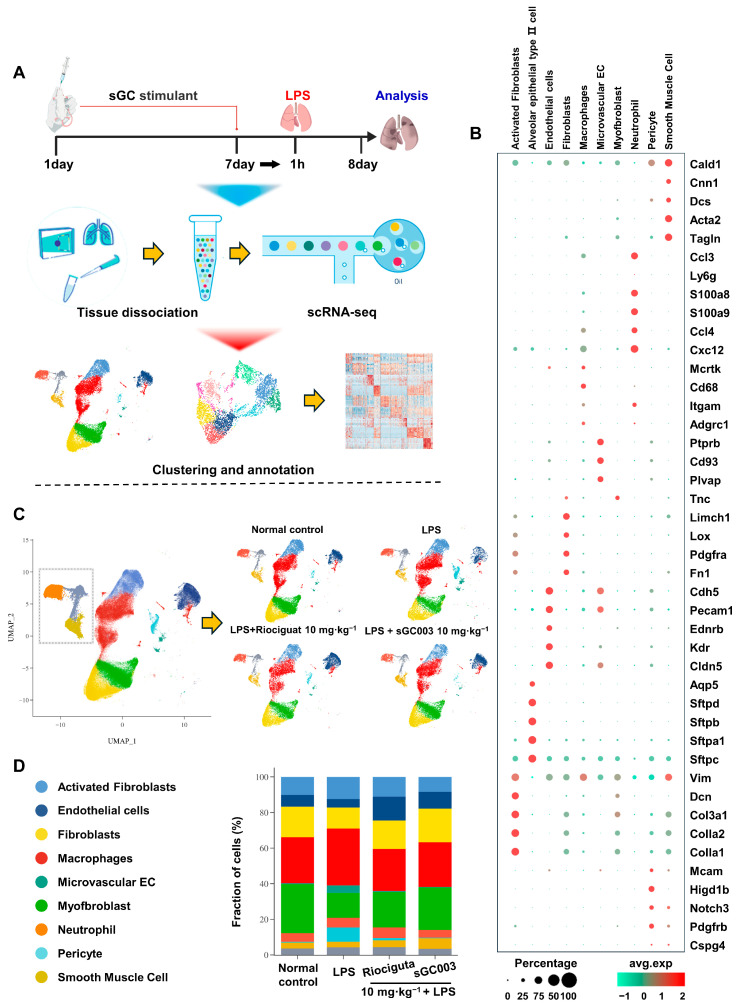
Single-cell RNA sequencing analysis and annotation of lung tissues from ALI mice. (**A**) Schematic overview depicting the approach for the experiments. (**B**) A dot plot displaying conserved marker genes in each cell population. (**C**) UMAP of cells from lung of mice in different treatment groups, revealing 10 different cell populations. (**D**) The fraction of cells from lung of mice in different treatment groups.

To investigate the mechanism of sGC003 in ALI treatment in mice, single-cell suspensions of mouse lung tissues were obtained and subjected to single-cell transcriptome analysis (Figure 3A). Through the analysis of conserved marker gene expression profiles, ten major cell types were identified. Subsequently, four cell groups were delineated, and uniform manifold approximation and projection (UMAP) were performed (Figure 3B,C). In the LPS group of mice, microvascular ECs and pericytes exhibited decreased proportion, while fibroblasts, neutrophils, and macrophages showed increased proportion, various other lung wall cells displayed varying degrees of alterations. Following treatment with the sGC stimulator, the proportions of each cell population tended towards normal levels (Figure 3D).

### 2.4. NO-sGC-Cyclic Guanosine Monophosphate Signaling Between ECs and Pericytes Is Restored by sGC003 in Mice with ALI

**Figure 4:**
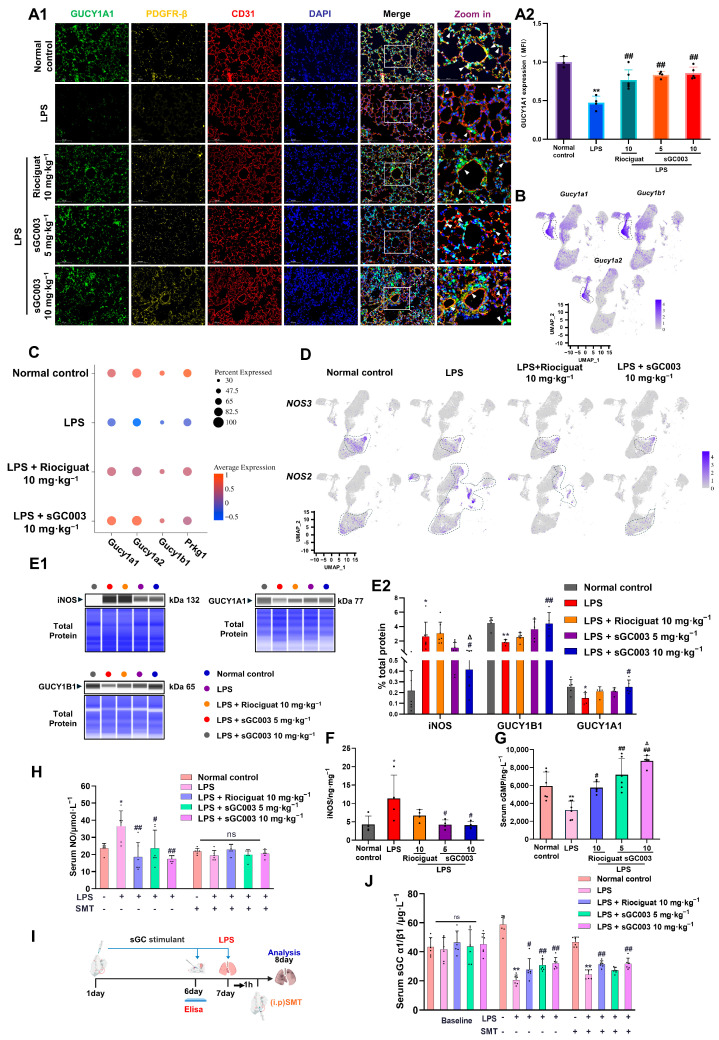
The NO-sGC-cGMP signaling pathway is stimulated by sGC003 in mice with ALI. (**A1**) Representative fluorescent images of GUCY1A1, PDGFR-β, and CD31 staining in lung sections. Scale bar: 100 μm (five columns on the left) and 50 μm (column on the right) (**A2**) The GUCY1A1 semiquantitative results of F1, *n* = 3–6. (**B**) The expression patterns for *Gucy1a1*, *Gucy1b1*, and *Gucy1a2*. (**C**) The dot plot displays the expression levels of sGC related genes in different groups of pericytes. (**D**) *NOS2*, *NOS3* expressing cells in all lung cells. (**E1**) Protein levels of iNOS, GUCY1A1, and GUCY1B1 validated by Simple Jess analysis. (**E2**) The semiquantitative results of (**E1**), *n* = 5–7. (**F**) Quantification of iNOS in mouse lung tissue, *n* = 4. (**G**) cGMP content in the serum of mice with ALI, *n* = 6. (**H**) NO levels in the serum of LPS and LPS + SMT mice, *n* = 6. (**I**) Schematic diagram illustrating the SMT experiment. (**J**) Quantification of sGC in mouse serum before and after LPS treatment, and quantification of sGC in mouse serum treated with SMT + LPS, *n* = 6. White arrows indicate colocalization regions. Data are presented as means ± SD. # *p* < 0.05, ## *p* < 0.01, vs. LPS group; * *p* < 0.05, ** *p* < 0.01, vs. Normal control group; Δ *p* < 0.05 vs. Riociguat group, ns means not significant.

Notably, pericytes were pinpointed as the target cells for sGC003 through gene expression profiling of sGC subunits (*Gucy 1a1*, *Gucy 1a2*, *Gucy 1b1*) and immunofluorescence co-localization verification (Figure 4A1,A2,B). In the mouse ALI model, a disruption of the pericellular NO-sGC-cyclic guanosine monophosphate (cGMP) pathway was observed (Figure 4C). Nitric oxide, primarily synthesized and released by endothelial nitric oxide synthase (eNOS, *NOS3*) in endothelial cells, serves as the principal stimulator of sGC to regulate its function in vivo. During ALI, there was a decrease in the expression of *NOS3* in endothelial cells, possibly associated with the reduction in endothelial cell proportion. The upregulation of iNOS (*NOS2*) suggested that the decline in NO was not the cause of sGC decrease; conversely, the excessive accumulation of pathological NO resulted in sGC oxidation inactivation [31]. The findings indicate that soluble guanylate stimulators primarily enhance the transcriptional levels of sGC and cGMP-dependent protein kinase 1 (PKG1, *Prkg1*) by boosting sGC sensitivity to NO under pathological conditions, rather than by upregulating eNOS expression (Figure 4D). Western blotting indicated that, relative to the normal control group, the LPS group exhibited significantly reduced protein expression of guanylate cyclase 1 soluble subunits α1 (GUCY1A1) and β1 (GUCY1B1), accompanied by a marked increase in iNOS protein level. In comparison to the LPS group, sGC003 significantly elevated GUCY1A1 and GUCY1B1 protein levels while notably reducing iNOS protein levels in the lung tissue of ALI mice (Figure 4E1,E2). ELISA analysis revealed a significant reduction in iNOS expression in the lung tissue of mice treated with sGC003 10 mg·kg^−1^ compared to the LPS group (Figure 4F). Consistent with the findings from fluorescence imaging and Western blot analysis, ELISA results demonstrated a marked decrease in sGC levels in the serum of mice with ALI (Figure 4J). This reduction in sGC levels with elevated pathological NO levels (Figure 4H), leading to a significant decrease in cGMP levels. Treatment with the sGC activator resulted in a notable increase in serum cGMP levels (Figure 4G) and a decrease in pathological NO levels in the serum as NO availability decreased and iNOS levels diminished (Figure 4H). In mice treated with the iNOS inhibitor SMT (Figure 4I) [39], sGC003 5 mg·kg^−1^ failed to significantly increase sGC levels relative to the LPS group (Figure 4J). Under conditions where pathological NO levels remained unchanged (Figure 4H), sGC003 5 mg·kg^−1^ produced only a modest stimulatory effect on sGC (Figure 4J).

### 2.5. Reversal of the Phenotypic Transformation of Pericytes by sGC003

**Figure 5:**
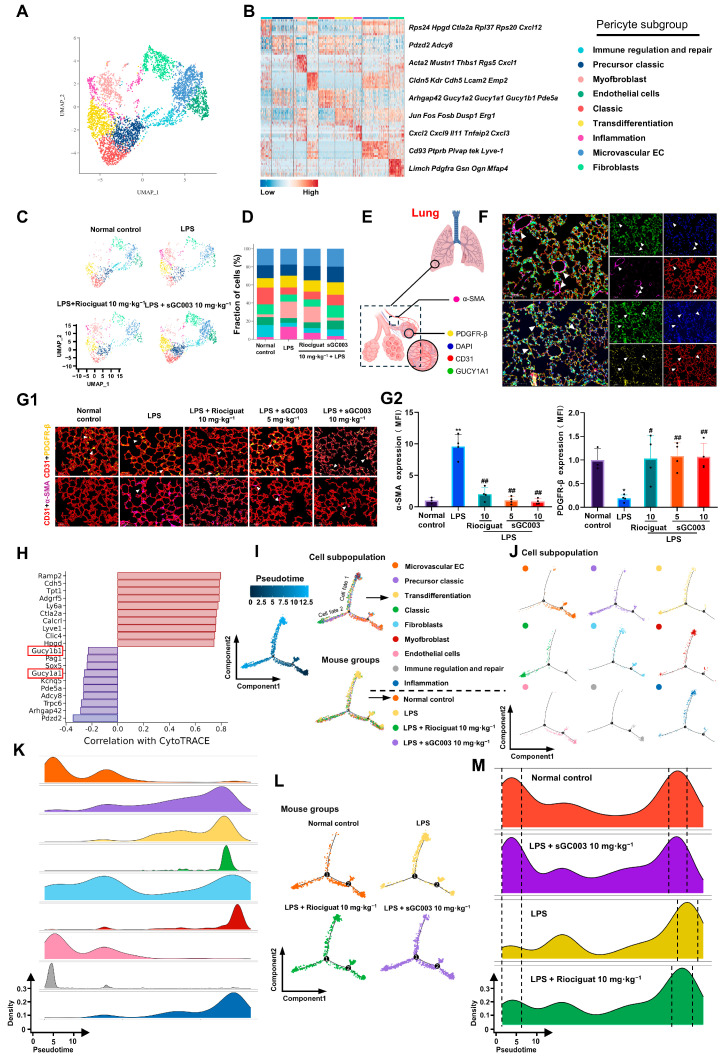
Soluble GC003 inhibits pericyte phenotypic transformation. (**A**) UMAP of pericyte subgroups. (**B**) Heatmap depicting the representative genes (ordered by adjusted *p*-values) of different subpopulation. (**C**) UMAP of different treatment groups. (**D**) The proportion of different cell subgroups in the lung cell populations of different treatment groups. (**E**) Schematic diagram of the expression sites of α-SMA and PDGFR-β in the lungs. (**F**) Localization of PDGFR-β and α-SMA in mouse lung tissue. Scale bar: 100 μm. (**G1**) Fluorescence images and fluorescence area quantification of PDGFR-β and α-SMA at the same location in lung tissue. Scale bar: 50 μm. (**G2**) The semiquantitative results of (**G1**), *n* = 3–4. (**H**) CytoTRACE analysis. (**I**) The differentiation trajectory of pericytes, with each color encoded as different subpopulations, treatment groups, and pseudotime. (**J**) Displays the differentiation trajectory of different subpopulations of pericytes using pseudotime. (**K**) The density plot displays the number of pseudo volume cells in the relative pseudotime scales of different subgroups. (**L**) Displays the different treatment groups of mice of pericytes using pseudotime. (**M**) The density plot displays the number of pseudo volume cells in the relative pseudotime scales of different treatment groups. The white arrows in (**F**) represent the colocalization of GUCY1A1, PDGFR-β, α-SMA, and CD31. White arrows indicate representative fluorescent regions of the protein. Data are presented as means ± SD. # *p* < 0.05, ## *p* < 0.01, vs. LPS group; * *p* < 0.05, ** *p* < 0.01, vs. Normal control group.

To investigate alterations in pericytes during ALI, transcriptomic data from all pericytes was extracted for subsequent analysis. Utilizing UMAP visualization (refer to Figure 5A), pericytes were classified into nine distinct subgroups, with the aid of heatmaps depicting differentially expressed genes:

- Immunoregulatory repair pericyte subgroup, characterized by high expression levels of cytotoxic T-lymphocyte associated protein 2α (*Ctla2a*), C-X-C motif chemokine ligand 12 (*Cxcl12*), and ribosomal proteins (*Rps24*, *Rpl37*, *Rpl20*), as well as others immune regulation and repair related genes.
- Precursor classical pericyte subgroup, exhibits a gene expression profile akin to classical pericytes, with notable upregulation of specific genes.
- Myofibroblast pericyte subgroup, characterized by high expression levels of alpha smooth muscle actin (*Acta2*), musculoskeletal embryonic nuclear protein 1 (*Mustn1*), thrombospondin-1 (*Thbs1*), and other genes associated with myofibroblast function.
- Endothelial cells (EC) pericyte subgroup, exhibit high expression levels of genes related to vascular endothelial function, such as Claudin 5 (*Cldn5*), kinase insert domain receptor (*Kdr*), and Cadherin 5 (*Cdh5*).
- Classical pericyte subgroup, identified by their high expression of pericyte-specific genes including Rho GTPase activating protein 42 (*Arhgap42*), *Gucy1a2*, *Gucy1a1*, and *Gucy1b1*.
- Transdifferentiation pericyte subgroup, characterized by high expression of genes like Jun proto-oncogene (*Jun*), Fos proto-oncogene (*Fos*), FosB proto-oncogene (*Fosb*), and other transdifferentiation-related genes.
- Inflammatory pericyte subgroup demonstrates high expression levels of genes associated with inflammatory responses, such as chemokines (*Cxcl2*, *Cxcl9*), interleukin 11 (*IL11*), and tumor necrosis factor α-induced protein 2 (*Tnfaip2*).
- Microvascular ECs pericyte subgroup, exhibits high expression levels of microvascular endothelium-related genes, including *CD93*, protein tyrosine phosphatase receptor type B (*Ptprb*), and plasma vesicle-associated protein (*Plvap*) and other Microvascular EC-related genes.
- Fibroblast pericyte subgroup exhibits elevated expression of fibroblast-associated genes such as LIM and calponin homologous domains containing 1 (*Limch1*), platelet-derived growth factor receptor α (*Pdgfra*), gelsolin (*Gsn*), and other fibroblast-related genes (Figure 5B).

Subsequently, the four groups of cells were visualized by UMAP (Figure 5C). A small number of myofibroblast pericyte subgroups were observed in the normal control group. The proportion of myofibroblast, inflammatory and transformed pericytes increased and the proportion of classical pericytes decreased in the LPS model group. sGC003 inhibited this transformation (Figure 5D). Immunofluorescence staining of the lungs for co-localization of α-SMA and CD31 confirmed that PDGFR-β mainly labels pericytes and α-SMA mainly labels vascular smooth muscle cells and myofibroblasts in healthy mice, whereas pericytes exhibited little to no α-SMA expression (Figure 5E,F). Using α-SMA, PDGFR-β, and CD31 immunofluorescence staining, we observed a significant decrease in the coverage area of PDGFR-β^+^ pericytes in the model group accompanied by a significant increase in α-SMA^+^ pericytes. Treatment with sGC003 significantly increased the coverage rate of PDGFR-β^+^ pericytes and inhibited the phenotypic transformation of pericytes to α-SMA^+^ compared with the ALI group (Figure 5G1,G2). Analysis using CytoTRACE revealed that highly differentiated cells (blue) exhibited elevated expression of genes linked to classical pericyte subgroups, while less differentiated cells (red) showed increased expression of genes associated with endothelial pericyte subgroups (Figure 5H). To investigate the link between pericyte and myofibroblast differentiation in ALI, Monocle2 pseudotime analysis was conducted on samples (Figure 5I), Migration trajectories of cells exhibited notable clustering on Pseudotime plots. Initially (Pseudotime 0–2.5), endothelial pericytes and immunoregulatory pericytes were prevalent, alongside some fibroblast pericytes. Subsequently (Pseudotime 2.5–5), classical pericytes emerged, and substantial accumulation of fibroblast pericytes. In the following stage (Pseudotime 5–7.5), endothelial pericytes and precursor classical pericytes clustered along pseudotime trajectories. By the fourth stage (Pseudotime 7.5–10), transitional pericytes and inflammatory pericyte subgroups were observed to emerge in substantial numbers and progressively align along Cell Fate 1 clusters. Concurrently, precursor classical pericytes occupied a predominant position within Cell Fate 2. At the terminal segment of the pseudotime trajectory (Pseudotime 10.0–12.5), classical pericytes, myofibroblastic pericytes, and inflammatory pericyte subgroups are abundantly aggregated (Figure 5J). Additionally, Figure 5K indicated a progression in the peak density of pericyte subgroups across different treatment groups over pseudo-timing. Notably, in LPS-induced ALI model mice, myofibroblast pericyte subgroups emerged as the primary differentiation trajectory for lung pericytes compared to the normal control group, a process effectively reversed by sGC003 (Figure 5L,M).

**Figure 6:**
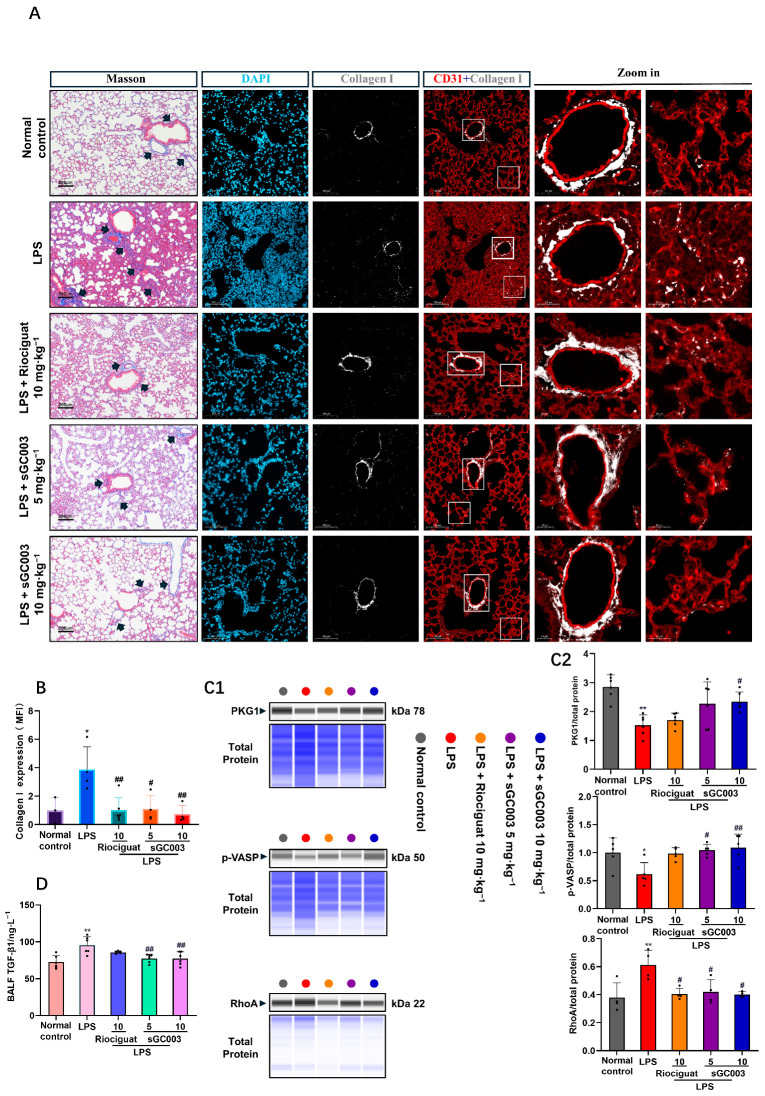
NO-sGC signaling stabilizes the microvascular environment by inhibiting RhoA-dependent cytoskeletal rearrangement through PKG1 phosphorylation of VASP. (**A**,**B**) Masson staining and fluorescence quantification of collagen fibers and collagens in lung tissue. Scale bar: 200 μm (Masson). Scale bar: 100 μm (three columns on the left); 20 μm (two columns on the right), *n* = 3–6. (**C1**) The PKG1, p-VASP, and RhoA protein levels were validated with Simple Jess analysis. (**C2**) The semiquantitative results of (**C1**), *n* = 4–6. (**D**) Quantification of TGF-β1 in bronchoalveolar lavage fluid (BALF), *n* = 6. Black arrows indicate a representative area of collagen fibers. Data are presented as means ± SD. # *p* < 0.05, ## *p* < 0.01, vs. LPS group; * *p* < 0.05, ** *p* < 0.01, vs. Normal control group.

To observe the effects of pericyte phenotypic transformation, we localized and quantified intrapulmonary collagen fibers and collagen. Masson staining showed that collagen fibers were mainly present in the blood vessels near the airways. In the ALI model group, the alveolar septa of the vascular walls of mice were significantly thickened, and collagen fibers presented as deep blue strands. Co-localization of collagen I and CD31 indicated that collagen in the alveolar septa was mainly present near microvessels (Figure 6A). Treatment with sGC003 prevented the increase in collagen fibers and collagen deposition caused by ALI (Figure 6B), possibly because sGC inhibited the transformation of pericytes into myofibroblasts. PKG 1 and p-vasodilator-stimulated phosphoprotein (p-VASP) decreased significantly and ras homolog family member a (RhoA) increased significantly in the model group. Soluble GC activated the downstream PKG 1, resulting in VASP phosphorylation at Ser 239 and downregulation of cytoskeletal RhoA expression. These effects of sGC003 prevented cytoskeletal rearrangement in pericytes (Figure 6C1,C2). In the BALF, sGC003 significantly reduced TGFB1 expression, which may be a key factor in inhibiting the phenotypic transformation of pericytes (Figure 6D). Furthermore, apoptosis of pulmonary microvascular endothelium in mice with ALI was determined using immunolocalization of cleaved caspase-3 and CD31. Only a few apoptotic cells were observed in control lungs. However, strong infiltration of cells with cleaved caspase-3 was observed in the lungs of mice with ALI. The sGC stimulator effectively prevented apoptosis of microvascular ECs (Figure S1A1,A2).

### 2.6. Activation of sGC Signaling Inhibits ALI-Induced Inflammation

**Figure 7:**
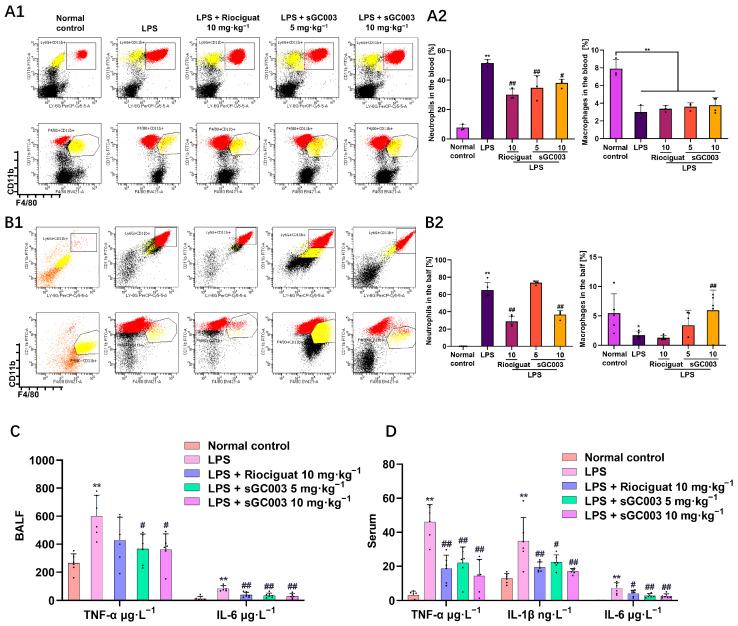
Blood inflammation and alveolar exudation are reduced by sGC003 in mice with ALI. (**A1**) FCM detection of relative levels of neutrophils and macrophages in mouse blood. (**A2**) The quantitative results of (**A1**), *n* = 3–4. (**B1**) FCM detection of relative content of neutrophils and macrophages in bronchoalveolar lavage fluid (BALF) of mice. (**B2**) The quantitative results of (**B1**), *n* = 4–5. (**C**) Inflammatory cytokine levels in the serum of mice with ALI, *n* = 6. (**D**) Inflammatory cytokine content in the BALF of mice with ALI, *n* = 6. Data are presented as means ± SD. # *p* < 0.05, ## *p* < 0.01, vs. LPS group; * *p* < 0.05, ** *p* < 0.01, vs. Normal control group.

**Figure 8:**
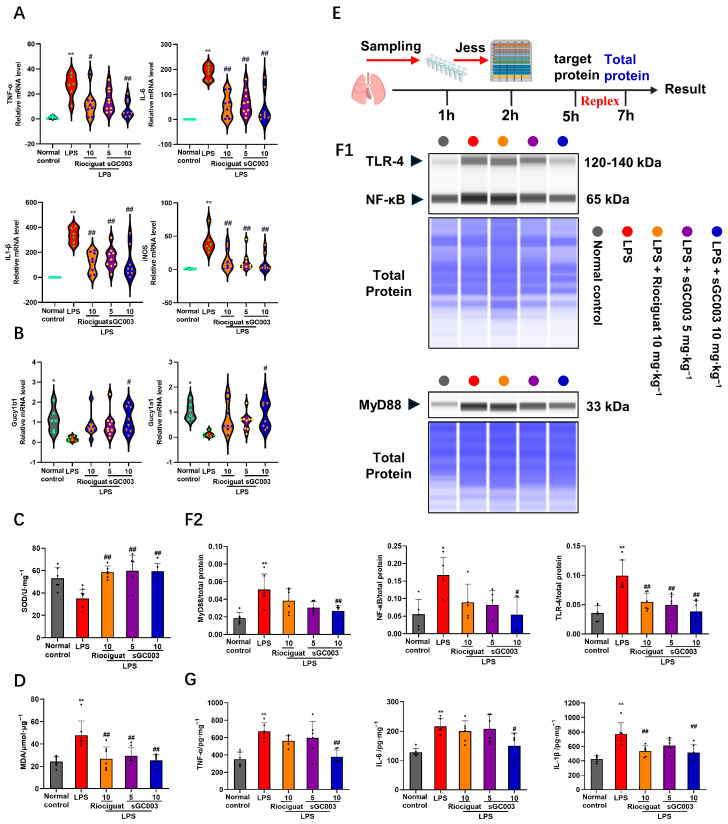
The expression of inflammatory cytokines in the lungs is reduced by sGC003 via inhibition of the TLR-4-MyD88-NF-κB-pathway. (**A**) Quantitative PCR validation of inflammatory cytokine expression in whole lung lysates from mice with ALI, *n* = 6–9. (**B**) Quantitative PCR validation of *Gucy1a1* and *Gucy1b1* in whole lung lysates from mice with ALI, *n* = 6–9. (**C**) Changes in SOD content in mice with ALI, *n* = 6. (**D**) Changes in MDA content in mice with ALI, *n* = 7. (**E**) Schematic diagram illustrating the operation process of Simple Jess. (**F1**) The protein levels of TLR-4, MyD88, and NF-κB were validated by Simple Jess analysis. (**F2**) The semiquantitative results of (**F1**), *n* = 5–6. (**G**) Determination of inflammatory cytokine content in lung tissue of mice with ALI, *n* = 6. Data are presented as means ± SD. # *p* < 0.05, ## *p* < 0.01, vs. LPS group; * *p* < 0.05, ** *p* < 0.01, vs. Normal control group.

The experiments above demonstrated that sGC003 can reverse dysfunctional signaling pathways induced during ALI and regulate pericytes to reduce vascular leakage. Thus, sGC003 may exert anti-inflammatory effects by modulating the pericyte phenotype. Flow cytometry analysis of neutrophils and macrophages in the blood and BALF revealed that sGC stimulants significantly reduced neutrophil levels in both blood and BALF; Notably, sGC003 significantly reduced macrophage migration in BALF (Figure 7A1,A2,B1,B2). The sGC stimulants markedly reduced inflammation levels in mice (Figure 7C,D). Jess assay analysis of lung lysates, (Figure 8E) revealed that sGC003 significantly reduced the expression of toll-like receptor 4 (TLR-4) and the downstream myeloid differentiation primary response 88-nuclear factor kappa-light-chain-enhancer of activated B cells (MyD88-NF-κB) signaling pathway (Figure 8F1,F2). These effects of sGC003 may be due to the stabilization of endothelial cell–pericyte interactions. ELISAs and qPCR analyses of the lung lysates showed that sGC003 also significantly decreased the transcription of inflammatory factors in the tissue (Figure 8A,G). Notably, *Gucy1a1* and *Gucy1b1* mRNA levels were significantly reduced in the LPS group, and sGC003 significantly increased the mRNA expression of *Gucy1a1* and *Gucy1b1* compared with the LPS group, validating the conclusions of the previous experiments (Figure 8B). NO is a key factor in the oxidative stress response, affecting superoxide dismutase (SOD) and malondialdehyde (MDA) levels. SOD expression is significantly enhanced and MDA levels are reduced by sGC003 due to the increased bioavailability of NO and the decreased pathological NO levels (Figure 8C,D).

## 3. Discussion

In ALI animal models and patients, increased permeability of the alveolar–capillary barrier to fluids and inflammatory cells leads to acute pulmonary edema, which is a major cause of mortality in ALI patients [40,41]. To date, no specific pharmacological treatments have been developed to treat ALI. However, a deeper understanding of the mechanisms underlying pulmonary edema has led to the development of numerous agents to reduce vascular permeability in patients with ALI [4,42].

We found that sGC003 exhibits outstanding efficacy in repairing sGC signaling in damaged EC pericytes. Compared with riociguat, sGC003 has lower energy in the binding process with sGC, forms the main conformation faster, and binds with more residues through hydrogen bonds, indicating a more stable binding between sGC003 and sGC. Thus, sGC003 significantly slows the development of LPS-induced ALI, maintains vascular integrity, prevents phenotypic changes in pericytes, reduces damage to endothelial cells, and attenuates vascular leakage. Like riociguat [43], sGC003 specifically binds to the gap between the β H-NOX domain and CC domain of sGC, improving the utilization of NO by sGC under pathological conditions and significantly increasing downstream cGMP production. Increased cGMP production induced by sGC003 increases microvascular stability and leads to strong anti-inflammatory activity and protective effects.

Pulmonary vascular wall cells include pericytes and smooth muscle cells. Most pericytes surround the alveolar capillaries [44,45]. Single-cell transcriptome and immunofluorescence co-localization experiments demonstrated that sGC is predominantly localized to pericytes, sGC003 can specifically activate the NO-sGC signaling pathway in ALI models. The pseudotime analysis of pericyte subgroups revealed that during the initial phase, pulmonary vessels recruited pericytes associated with vascular endothelium, which expressed numerous vascular endothelium-related genes (*Cldn5*, *Cdh5*, *Kdr*, *Plvap*, *CD93*, *Ptprb*) to establish capillary networks. Subsequently, along the pseudotime trajectory, pericytes underwent significant gene rearrangements, including *Arhgap42*, *Gucy1a1*, *Gucy1a2*, *Gucy1b1*, and *Cspg4*, leading to the suppression of vascular endothelium-related gene expression, indicating the onset of pericyte maturation. This maturation process is precisely regulated by PDGF-BB, a key recruitment factor secreted by endothelial cells [46,47]. PDGF-BB binding to PDGFR-β on the pericyte surface promotes pericyte migration, proliferation, and differentiation toward the vessel wall, enhances their signal responsiveness, and ultimately drives their maturation into vessel-covering pericytes [48]. This process is essential for establishing a competent endothelial barrier. However, in mice experiencing ALI, inflammation or vascular injury reduces PDGFR-β expression disrupts the interaction between microvascular ECs, causing a substantial subset of pericytes to transition from vascular endothelium-associated or classical pericytes to inflammatory or altered pericytes, ultimately differentiating into myofibroblasts and expressing myofibroblast-related genes such as *Acta2*, *Mustn1*, and *Thbs1*. These findings suggest that fluid leakage in the lung tissue of mice in an ALI model originates from two primary factors: firstly, the compromised vascular barrier function due to reduced pericellular coverage of mature capillaries, and secondly, the structural abnormalities in the newly formed vascular network, which fails to establish a complete endothelial support system due to pericyte transformation or recruitment disorder. However, sGC stimulators reversed this change, supporting the critical role of the NO–sGC axis in promoting pericyte recruitment and functional vascular maturation. Related studies further confirm that increasing local sGC sensitivity to NO effectively drives pericyte recruitment and maturation, thereby promoting the stabilization and functional maturation of neovascularization [49].

Recent research on fibrotic scars and fibroblast origins showed that changes in pericyte phenotype directly impact vascular homeostasis and disease progression [50]. Investigations of cytoskeletal-associated genes reveal that inflammation activates the cytoskeleton-associated gene RhoA, leading to the interconversion of F-actin and G-actin [6,51,52]. Arhgap42 functions as a negative regulator of RhoA, and its downregulation or loss represents a major factor driving elevated RhoA activity [53]. The cytoplasmic protrusions of pericytes contract, resulting in the rearrangement of the pericyte cytoskeleton and detachment from vascular endothelial cells [3,54]. Researchers have observed that abnormal contraction of pericytes leads to a significant upregulation of α-SMA expression [55,56], likely resulting from enhanced formation of actin stress fibers induced by Rho activation [57,58]. sGC stimulators attenuate pericyte-to-myofibroblast transition by modulating pericyte subpopulation differentiation, upregulating Arhgap42 expression in pericytes, and activating PKG-mediated phosphorylation of VASP at Ser239 [59], thereby inhibiting RhoA activity. TGF-β1 is a potent inducer of pericyte phenotype transformation [60]. When pericytes are stimulated by LPS, the abnormal activation of the TLR-4/MyD88/NF-κB pathway and the excessive expression of inflammatory factors induce cell damage and increased TGF-β1, manifested as the down regulation of PDGFR-β and increased α-SMA expression [61,62,63,64,65]. Flores-Costa et al. demonstrated that PKG-mediated phosphorylation of VASP at Ser239 reduces NF-κB activity and the expression of interleukin-1β (IL-1β) [66], which is consistent with the downregulation of TLR-4/MyD88/NF-κB signaling by sGC003 in the ALI model. The induction of anti-fibrotic effects through sGC-cGMP-dependent pathway inhibit TGF-β1 signaling [67,68,69], promote collagen degradation gelatinases (MMP-2 and MMP-9) [70], and reduce the expression of TGF-β1 through non-classical pathways [71].

sGC003 exhibits anti-inflammatory effects and improves oxidative stress. In physiological conditions, NO serves as the most potent activator of sGC to modulate vascular homeostasis; in murine models of ALI, endothelial injury causes pathological NO release due to excessive iNOS expression, leading to sGC heme oxidation and catalytic inactivation [72]. This results in decreased NO utilization and pathological accumulation of NO, which reacts with superoxide anions to form peroxynitrite, exacerbating oxidative stress [73,74]. Soluble GC stimulation with sGC003 reduces pathological NO and increases NO utilization, thereby improving the SOD/MDA ratio and mitigating oxidative stress and, ultimately, protecting the organism.

## 4. Materials and Methods

### 4.1. Mouse Experiments

SFR grade ICR male mice, weighing 25 ± 2 g and aged 6–8 weeks, were obtained from Beijing Hua fu Kang Biotechnology Co., Ltd. (SCxK, Beijing, China, 2019-0008). Mice were housed in the animal facility at the Military Medical Academy in Beijing under controlled conditions, including a temperature of 22 ± 2 °C, humidity of 50 ± 10%, and a 12 h light/dark cycle. The mice had ad libitum access to standard chow and water. Mice were acclimatized for at least 7 days before the beginning of the experiments.

### 4.2. Material

Detailed information on manufacturers and catalog numbers is provided in the Supplementary Data.

### 4.3. Pharmacological Treatment Regimen

Mice were randomly divided into the following five treatment groups: LPS + riociguat group, 10 mg·kg^−1^ of riociguat solution (positive control); LPS + sGC003 group, 5 mg·kg^−1^ of sGC003 solution and LPS + sGC003 group, 10 mg·kg^−1^ of sGC003 solution; normal control and LPS groups of riociguat and sGC003 solution, equal volume of solvent. Prior to modeling, Riociguat and sGC003 were administered gavage to mice daily over a 7-day period. The normal control group and LPS group were administered 1% DMSO in physiological saline daily. The ALI model was established in the mice one hour after the final dose on the seventh day. Before oral gavage, the sGC003 was dissolved in 1% DMSO and mixed with 99% of 0.9% NaCl to prepare a fresh drug solution.

### 4.4. Intratracheal LPS Instillation-Induced Lung Injury Model

We anesthetized the mice with pentobarbital. When the mice began to breathe primarily through their mouths, the noses were clamped with tweezers, and a micropipette was used to instill either 0.9% NaCl or 0.9% NaCl containing LPS (4 mg/kg) into the throat to induce ALI. The mice were shaken for a few seconds to promote liquid dispersion. After 24 h, the mice were euthanized after spontaneous activity test, pulmonary function test, micro-CT evaluated pulmonary status, and periorbital blood was collected. The thoracic cavity was opened to expose the lungs, and the left lung was ligated at the hilum. The trachea was exposed and instilled with phosphate-buffered saline (PBS) that had been pre-chilled on ice. After the lungs were fully inflated, the fluid was withdrawn. This procedure was repeated three times. The final sample was collected, targeting a recovery rate of approximately 80%. Subsequently, the left lung was removed for wet/dry ratio measurement, the right upper lobe was fixed with 4% paraformaldehyde, and the remaining lung lobes were stored at −80 °C for subsequent analysis.

### 4.5. Spontaneous Activity Test

Spontaneous activity tests were performed in the morning under enclosed, stimulus-free conditions, with all groups run in parallel. Spontaneous activity serves as an indicator of physiological state and disease impact. Before formal testing, the activity chambers were cleaned, and mice were allowed a 2 min habituation period. Then, exploration activity was assessed for 10 min using a Shanghai Jiliang (Shanghai, China) general activity box. Data were analyzed using the built-in system.

### 4.6. CT Examination

Micro-computed tomography (CT) was used to assess the lung condition in mice (Micro-CT Skyscan 1276, Bruker Co., Ltd., Karlsruhe, Germany). CT scans were performed 24 h after LPS treatment. Mice were anesthetized with isoflurane and fixed in the micro-CT scanner with the following parameters for chest imaging: Source Voltage 50 kV, Source Current 200 µA, and voxel size 35 × 35 × 35 µm^3^.

### 4.7. Pulmonary Function Test

Lung function in mice was assessed by whole-body plethysmography using a WBP-8MR system (TOW Co., Ltd., Shanghai, China). For three consecutive days prior to measurement, mice were housed in the testing chamber for 30 min daily to acclimate to the environment. On the test day, the area was kept quiet and free of airflow disturbances to maintain controlled conditions. Following a 30 min habituation period in the chamber, each mouse was recorded for over 20 min. Data from the final 10 min of the 20 min recording were averaged for analysis, and plethysmography curves were generated using the manufacturer’s software (Version ResMass 2.7.2, TOW Co., Ltd., Shanghai, China).

### 4.8. Lung Wet/Dry Weight Ratio

The left lungs were harvested and immediately weighed. After drying the lungs in an 80 °C oven, the dry weights were measured. The lung W/D (wet/dry) ratio was calculated by dividing the wet weight by the dry weight.

### 4.9. Lung Tissue Staining with Hematoxylin and Eosin (H&E)

Following fixation in 4% paraformaldehyde for over 24 h, mouse lungs were harvested, dehydrated, sectioned, and subjected to H&E staining. Detailed experimental procedures are provided in the Supplementary Materials.

### 4.10. Evans Blue Detection of Lung Permeability in Mice

Evans blue dye (50 mg/kg) was injected intravenously into the tail vein. After 1 h, mice were euthanized with an overdose of pentobarbital. The thoracic cavity was opened, the left atrium was cut with ophthalmic scissors, and the pulmonary vasculature was flushed through the right ventricle with saline until the effluent became clear. Lungs were then removed, immediately weighed to obtain wet weight, and approximately 100 mg of lung tissue was collected, weighed, and transferred to a tube with 1 mL of formamide. The tissue was homogenized and incubated at 60 °C for 24 h. Following centrifugation at 12,000× *g* for 15 min, the supernatant was taken, and its absorbance was measured at 610 nm using a standard Evans blue curve to determine dye extravasation. Evans blue leakage per gram of lung tissue was calculated as the dye concentration divided by the tissue wet weight.

### 4.11. Molecular Docking

Molecular docking of riociguat and sGC003 with the sGC protein was performed using AutoDock Vina 1.2.3, and visualization was carried out with PyMOL 2.5.2 and Maestro. Detailed experimental procedures are provided in the Supplementary Materials.

### 4.12. Molecule Dynamics Simulation

Molecular dynamics simulations were performed using AMBER 24 [75,76,77,78,79,80,81,82,83,84]. Detailed experimental procedures are provided in the Supplementary Materials.

### 4.13. MM/GBSA Binding Free Energy Calculation

The binding free energy for each protein–ligand complex was calculated with the MM/GBSA method [85,86,87,88,89]. Detailed experimental procedures are provided in the Supplementary Materials.

### 4.14. Principal Component Analysis

The 500 ns simulation trajectory was preprocessed using the cpptraj module in AmberTools, including removal of solvent and ions, autoimaging, and RMS fitting based on protein Cα atoms. An average structure was then generated as reference, with all trajectory frames aligned to this reference. Subsequently, the covariance matrix of positional fluctuations for non-hydrogen atoms was calculated and subjected to eigen decomposition to obtain eigenvectors and eigenvalues. The first 20 eigenvectors were retained, with PC1 and PC2 extracted for further analysis. Finally, the projection of the trajectory onto PC1 and PC2 was used to construct the free energy landscape (FEL).

### 4.15. Single-Cell RNA Sequencing and Data Analysis

Except for the LPS + sGC003 5 mg·kg^−1^ group, lung cells from three mice per group were pooled and loaded onto a 10 × Chip S3 single-cell chip. The SeekOne^®^ DD Single Cell 3′Transcriptome Library Construction Kit, SeekOne^®^ DD Single Cell 3′ Reverse Transcription Kit, and SeekOne^®^ DD Single Cell 3’Microbead Kit (SeekGene Co., Ltd., Beijing, China) were employed following the manufacturer’s protocols and sequenced on Illumina (Illumina and Huada platforms) and Huada (MGI Tech Co., Ltd., Shenzhen, China) platforms. Data analysis was conducted using SeekOne^®^ Tools, a software developed by SeekSoul (Version 5.2.1.250415, SeekGene Co., Ltd., Beijing, China), with cell development trajectories inferred using Monocle2 (Version 2.22.0, Trapnell Laboratory at the Broad Institute, Seattle, WA, USA).

### 4.16. Immunofluorescence Staining

Immunofluorescence staining was performed on sections prepared from the paraffin blocks described in Section 4.10, with results examined under a fluorescence microscope (Eclipse E100, Nikon Corporation, Tokyo, Japan) and analyzed using ImageJ software (Version 1.53t, National Institutes of Health, Bethesda, MD, USA). Detailed experimental procedures are provided in the Supplementary Materials.

### 4.17. Jess Capillary-Based Electrophoresis Immunoblot Assays

Lung tissue was lysed in RIPA buffer without SDS and supplemented with phosphatase and protease inhibitors. After measuring protein concentrations using a BCA protein assay, Western blotting was performed with the Jess automated Western blot system (Jess, ProteinSimple Inc., San Jose, CA, USA). Capillary electrophoresis was performed using a 12–230 kDa separation capillaries. Antibodies were diluted 1:50. Data were quantitatively analyzed using Jess Compass for SW software (Version 6.3.0, ProteinSimple Inc., San Jose, CA, USA) [90,91,92]. Detailed experimental procedures are provided in the Supplementary Materials.

### 4.18. Enzyme Linked Immunosorbent Assay (ELISA)

The mice were fasted one night before sampling. After fasting for 12 h, the mice were anesthetized and blood was taken from their eyeballs. Bronchoalveolar lavage fluid (BALF) and lung tissues were taken out according to the method in 2.5 of Part II. The peripheral blood was allowed to stand at room temperature for 4 h and then centrifuged at 1000× *g* for 20 min to take out the upper serum. The lung tissues were extracted and homogenized. After BCA, BALF was immediately centrifuged to take out the supernatant. The experimental operation was carried out strictly according to the instructions of the ELISA kit to detect the corresponding indicators. Detailed experimental procedures are provided in the Supplementary Materials.

### 4.19. S-Methylisothiourea Sulfate Treatment

After administering the drug for seven days, mice from each group received an intraperitoneal injection of 10 mg·kg^−1^ S-methylisothiourea sulfate [93] (SMT, iNOS and NOS2 inhibitor) one hour after LPS treatment. Periorbital blood was collected 24 h post-LPS treatment for analysis.

### 4.20. Quantitative Real-Time Polymerase Chain Reaction (qRT-PCR)

RNA was extracted from mouse lung tissue, according to the manufacturer’s instructions (Accurate Biology, Changsha, China). RNA concentration was quantified using the Nanodrop DS-500 (Thermo Fisher Scientific, Waltham, MA, USA). Genomic DNA was removed and reverse transcription was performed using a reverse transcription kit (AG11728, Accurate Biology, Changsha, China), with 1500 ng of total RNA in a 30 μL reaction volume. The reaction mixture was prepared, vortexed, and aliquoted into PCR plates. Three technical replicates were measured per gene for each sample. Amplification was conducted using 1 μL of cDNA on a QPCR instrument (Thermo Fisher Scientific, USA). GAPDH was used to normalize the measurements and data were analyzed using the 2^−∆∆CT^ method. The primer sequences for qRT-PCR are listed in Table 1.

### 4.21. Flow Cytometry (FCM) Analysis

Heparin sodium (0.8–1 mL) was added to orbital blood samples and incubated at room temperature. BD Leukocyte Activation Cocktail (4 μL) with BD GolgiPlug was mixed with anticoagulated whole blood (200 μL) and incubated at 37 °C in a 5% CO_2_ incubator or 37 °C water bath for 4–6 h. Samples were incubated with CD45, LY-6G, CD11b, and F4/80 antibodies at room temperature in the dark for 15–30 min before flow cytometry.

Bronchoalveolar lavage fluid was centrifuged to remove the supernatant, and the cells were stained with CD45, LY-6G, CD11b, and F4/80 antibodies at room temperature in the dark for 15–30 min before flow cytometry.

Data were analyzed using BD FACSDiva 8 software.

### 4.22. Statistical Analysis

All quantitative data are presented as means ± standard deviation (SD) and analyzed using Graph Prism 9.5 software (San Diego, CA, USA). Groups were compared with Student’s *t*-tests (two groups) or one-way analysis of variance (ANOVA; multiple groups). *p* < 0.05 was considered statistically significant.

## 5. Conclusions

**Figure 9:**
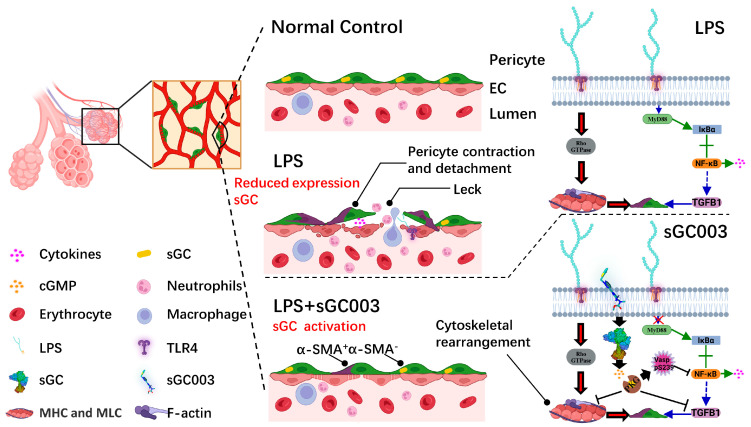
The schematic illustrates the essential role of the NO–sGC signaling pathway in preserving endothelial-pericyte stability and vascular integrity. Under LPS-induced ALI, NO–sGC signaling is impaired, resulting in cytoskeletal rearrangement and phenotypic switching of pericytes. This altered pericyte function leads to their detachment and elevated expression of inflammatory factors, which in turn promote immune cell infiltration, endothelial damage, and pulmonary edema. Treatment with the sGC stimulator sGC003 stabilizes endothelial–pericyte interactions, thereby helping to maintain vascular integrity and attenuate inflammation-driven lung injury.

Our results show that sGC003 reduces pathological NO and increases sGC activity, alleviates damage caused by oxidative stress, inhibits abnormal activation of the MyD88/NF-κB pathway, alters pericyte phenotype, and reduces the cell contraction caused by changes in the F-actin/G-actin ratio. We speculate that sGC003 directly inhibits the activation of TGF-β1 to reduce sGC expression and fibrosis caused by inflammation by inhibiting the TLR4/MyD88/NF-κB signaling pathway. In conclusion, sGC003 reduces the phenotypic transition of pericytes, stabilizes the interaction between pulmonary microvessels and pericytes, reduces the expression of inflammatory factors and the infiltration of inflammatory cells, and delays the progression of ALI to protect the lung from inflammation-induced injury. These results highlight the importance of pericytes to microvascular ECs and reveal the relevant target cells of sGC003 to reduce vascular leakage (Figure 9). In the future, sGC003 may provide a new drug regimen for the treatment of ALI.

## Figures and Tables

**Table 1:** Primer sequences for RT-qPCR.

Primer Name	Forward Primer (5–3′)	Forward Primer (5–3′)
qMouse TNF-α	CCCTCACACTCACAAACCAC	ACAAGGTACAACCCATCGGC
qMouse IL-6	AGCCAGAGTCCTTCAGAGAGA	GCCACTCCTTCTGTGACTCC
qMouse IL-1β	GCCACCTTTTGACAGTGATG	GAAGGTCCACGGGAAAGACA
qMouse GUCY1A1	CCAGATAGCACTGATGGCCC	GGGCATCTTCACTCCGACAA
qMouse GUCY1B1	CAATCGGGATCCATACCGGG	AGTGGATCCGAGTTTTCTGTATGT
qMouse iNOS	CTCGGAACTGTAGCACAGCA	GCACATCAAAGCGGCCATAG
qMouse GAPDH	GGAGCGAGATCCCTCCAAAAT	GGCTGTTGTCATACTTCTCATGG

## Data Availability

The original contributions presented in this study are included in the article/Supplementary Materials. Further inquiries can be directed to the corresponding authors.

## Supplementary Materials

The following supporting information can be downloaded at: https://www.mdpi.com/article/10.3390/ijms27031346/s1.
