# Supplementary material for: The Novel Soluble Guanylate Cyclase Stimulator Attenuates Acute Lung Injury via Inhibiting Pericyte Phenotypic Transition
Source: Int J Mol Sci. 2026 Jan 29;27(3):1346. doi: 10.3390/ijms27031346 (PMC12897666; doi:10.3390/ijms27031346)
Supplement: Supplementary file 1 [file ijms-27-01346-s001.zip › ijms-4049324-supplementary.pdf]

Supporting Information

Supplementary Figure

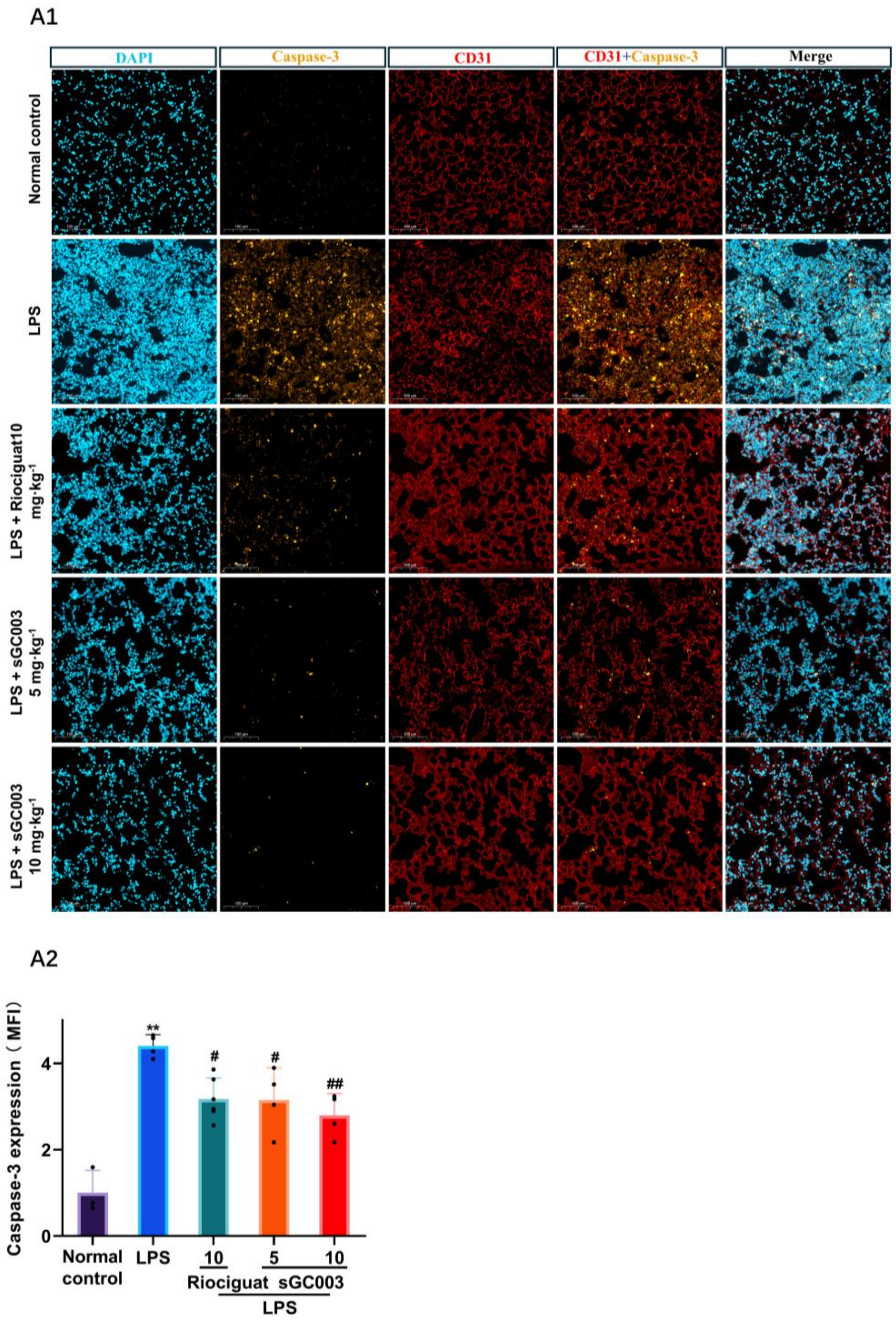

Supplementary figure S1. sGC003 inhibits apoptosis in lung tissue of ALI mice. **(A1)** Display representative fluorescent images of Caspase-3 and CD31 stained lung sections. Scale bar: 100μm. **(A2)** The semiquantitative results of A1. n=3-6. Data are presented as means ± SD. $^{\#}P < 0.05$, $^{\#\#}P < 0.01$, vs. LPS group; $^{*}P < 0.05$, $^{**}P < 0.01$, vs. Normal control group.

Supplementary materials and methods

1.1. Materials

Jess Automated Protein Expression and Quantitative Analysis System (ProteinSimple, USA); Frozen high speed centrifuge (eppendorf, Germany, Model: 5424R); Freezing grinding instrument (Beijing Hede Technology Co., LTD., model: Beijing N9548R) LPS (L2880, Sigma Chemical Company, St. Louis, MO, USA); Evans Blue (G1810, Beijing Solarbio Science & Technology, Beijing, China); mouse tissue IL-6 ELISA kit (SEKM-0007, Beijing Solarbio Science & Technology, Beijing, China); mouse tissue TNF-α ELISA kit (SEKM-0034, Beijing Solarbio Science & Technology, Beijing, China); Formamide (A100314-0500, Sangon Biotech, Shanghai, China); S-methylisothiourea sulfate (HY-79457, MedChemExpress, Newark, NJ, USA); mouse TNF-α ELISA kit (JL10484, Shanghai Jianglai Biotechnology, Shanghai, China); mouse IL-6 ELISA kit (JL20268, Shanghai Jianglai Biotechnology, Shanghai, China); mouse IL-1β ELISA kit (JL18442, Shanghai Jianglai Biotechnology, Shanghai, China); mouse sGC ELISA kits (JL47646, Shanghai Jianglai Biotechnology, Shanghai, China); mouse cGMP ELISA kits (CEA577Ge, Cloud-Clone, Wuhan, Hubei Province, China); mouse NO synthase (NOS) 2 ELISA kits (SEA837Mu, Cloud-Clone, Wuhan, Hubei Province, China); NO detection kits (A012-1-2, Nanjing Jiancheng, Bioengineering Institute, Nanjing, Jiangsu Province, China); SOD detection kits (S0101M, Beyotime Biotechnology, Shanghai, China); MDA detection kits (S0131M, Beyotime Biotechnology, Shanghai, China); Mouse TGF-β1 ELISA kit (MM-0135M1, Meimian, Yancheng, Jiangsu, Province, China). 4% paraformaldehyde (G1101, Servicebio, Wuhan, Hu-bei Province, China). DAPI (G1012, Servicebio, Wuhan, Hu-bei Province, China); PBS (G4202, Servicebio, Wuhan, Hubei Province, China); BCA kit (AR1097, Boster Bio, Pleasanton, CA, USA); Protease Inhibitor Cocktail 100× (CW2200S, Cwbio, Taizhou, Jiangsu Province, China); Phosphatase Inhibitor Cocktail 100× (CW2383S, Cwbio, Taizhou, Jiangsu Province, China).Rabbit anti-GUCY1B1 antibody (ab154841, Abcam, Boston, MA, USA); Rabbit anti-iNOS antibody (ab178945, Abcam, Boston, MA, USA); Fluores-cence-conjugated (FITC) mouse anti-gamma actin antibody (ab123034, Abcam, Boston, MA, USA); Mouse anti-PDGFR-β (FITC) antibody (ab69506, Abcam, Boston, MA, USA); Mouse anti-α-SMA (FITC) antibody (GB13044-50, Servicebio, Wuhan,

Hu-bei Province, China); Rabbit anti-CD31 (FITC) antibody (GB113151, Servicebio, Wuhan, Hu-bei Province, China); Rabbit anti-GUCY1A1 (FITC) antibody (orb214026, Bi-orbyt, Cambs, UK); Rabbit anti-TLR-4 antibody (#38519, Cell Signaling Technology, Danvers, MA, USA); Rabbit anti-NF-κB antibody (#8242, Cell Signaling Technology, Danvers, MA, USA); Rabbit anti -Myd88 antibody (#4283, Cell Signaling Technology, Danvers, MA, USA); Rabbit anti- PKG1 antibody (3248, Cell Signaling Technology, Danvers, MA, USA); Rabbit anti-p-VASP antibody (3114, Cell Signaling Technology, Danvers, MA, USA); Rabbit anti-RhoA antibody (10749-1-AP, proteintech, Wuhan, Hubei Prov-ince, China); Mouse anti-rat CD45 allophycocyanin (APC) (610266, Becton Dickinson Company, Franklin Lakes, NJ, USA); Rat anti-mouse LY-6G PerCP-Cy5.5 antibody (560602, Becton Dickinson Company, Franklin Lakes, NJ, USA); Mouse anti-CD11b (FITC) antibody (101206, BioLegend, San Diego, CA, USA); F4/80 Brilliant Violet 421 (123132, BioLegend, San Diego, CA, USA); Cy3 conjugated Donkey Anti-Rabbit IgG (GB21403, Servicebio, Wuhan, Hu-bei Province, China); Cy3 conjugated Donkey Anti-Mouse IgG (GB21401, Servicebio, Wuhan, Hu-bei Province, China).

1.3. Lung Tissue Staining with H&E

Dehydration: Fixed mouse lung tissues were dehydrated through a graded ethanol series (70%, 80%, 90%, 95%, 100%), immersing for 1–2 h per step; 2) Clearing and Paraffin Infiltration: Tissues were cleared in xylene and infiltrated with molten paraffin at 60 °C overnight; 3) Sectioning: Blocks were sectioned at 5–10 μm and mounted on slides; 4) Deparaffinization and Rehydration: Paraffin sections were deparaffinized in solutions I/II, then rehydrated through ethanol (100%, 75%); frozen sections were warmed, fixed (15 min), and rinsed; 5) Staining: Sections were stained with hematoxylin (3–5 min), differentiated, blued, rinsed, dehydrated (95% ethanol), and counterstained with eosin (15 s); 6) Dehydration and Mounting: Sections were dehydrated through ethanol, n-butanol, and xylene, then mounted with neutral balsam; 7) Microscopy: Slides were examined at 100× and 200× for inflammatory infiltration, hyaline membranes, hemorrhage, and septal thickening.

1.4. Molecular Docking

The 3D structure of the sGC protein receptor was obtained from the RCSB PDB database (http://www.rcsb.org/) using PDB ID 6JT2. Water molecules in the sGC crystal structure were removed using PyMOL 2.5.2, and all processed small molecules along with the receptor protein were converted into the PDBQT format required for AutoDock Vina 1.2.3 docking using ADFRsuite 1.0. KingDraW was used to draw the chemical structures of riociguat and sGC003, which were converted into accurate 3D structures using ChemDraw 19.0 followed by energy minimization with the MMFF94 force field. Finally, docking of riociguat and sGC003 with the sGC was performed using Autodock Vina 1.2.3 with the exhaustiveness of the global search set to 32 and other parameters retaining their default values, with the highest scoring output conformation considered as the bound conformation, and visualization and analysis of the docking results were performed using PyMOL 2.5.2 and Maestro.

1.5. Molecule Dynamics Simulation

Simulations were performed using AMBER 24. Prior to simulation, partial charges for small molecules were derived via the antechamber module with Hartree–Fock (HF) SCF/6-31G* calculations performed in Gaussian 09. HEM parameters were obtained from the literature reported by Kiumars Shahrokh et al. Small mol-ecules and the protein were described using the GAFF2 force field and the ff14SB force field, respectively. Each system was added hydrogen atoms with the LEaP module, add a truncated octahedral TIP3P water box at a distance of 10 Å, and add Na^+/Cl^- ions to balance the system charge, finally outputting the topology and parame-ter files for simulation.

First, the system underwent energy optimization, comprising 2500 steps of the steepest descent method followed by 2500 steps of the conjugate gradient method. Upon completion of energy optimization, the system was heated from 0 K to 298.15 K over 200 ps under constant volume and at a constant heating rate. While maintaining the temperature at 298.15 K, a 500 ps NVT (isothermal and isochoric) ensemble simula-tion was performed to achieve a more uniform distribution of solvent molecules within the solvent box. Subsequently, a 500 ps NPT (isobaric isothermal) equilibration simula-tion was conducted for the entire system. Finally, a 500 ns NPT (isobaric isothermal) ensemble

simulation was carried out for the complex under periodic boundary condi-tions. During the simulations, a nonbonded cutoff distance of 10 Å was used, long-range electrostatic interactions were calculated using the Particle Mesh Ewald (PME) method, bond lengths involving hydrogen atoms were constrained using the SHAKE algorithm and temperature was controlled using the Langevin algo-rithm with a collision frequency γ set to 2 ps^{-1}. The system pressure was maintained at 1 atm, the integration time step was 2 fs, and the trajectory was saved every 10 ps for subsequent analysis.

1.6. MM/GBSA Binding Free Energy Calculation

The binding free energy between the protein and ligand in all systems was calculated using the MM/GBSA method. In this study, MD trajectories from 450–500 ns were used for the calculation. The specific formula is as follows:

$$\Delta G_{bind} = \Delta G_{complex} - (\Delta G_{receptor} + \Delta G_{ligand}) = \Delta E_{internal} + \Delta E_{VDW} + \Delta E_{elec} + \Delta G_{GB} + \Delta G_{SA} \quad (1)$$

In Equation (1), ΔEinternal represents internal energy, ΔEVDW denotes van der Waals interactions, and ΔEelec signifies electrostatic interactions. The internal energy includes bond energy (Ebond), angle energy (Eangle), and torsional energy (Etorsion). ΔGGB and ΔGSA are collectively referred to as solvation free energy, where GGB corresponds to polar solvation free energy and GSA represents nonpolar solvation free energy. For ΔGGB, the GB model developed by Nguyen et al. was employed (igb = 2). The ΔGSA was calcu-lated as the product of surface tension (γ) and solvent-accessible surface area (SASA): ΔGSA = 0.0072 × ΔSASA. Entropic changes were omitted in this study due to high computational cost and low accuracy.

1.7 Immunofluorescence Staining

Slides were sequentially immersed in Environmental Dewaxing Solution I for 10 min, Environmental Dewaxing Solution II for 10 min, Environmental Dewaxing Solution III for 10 min, and Anhydrous Ethanol I, II, and III for 5 min each. Slides were rinsed with distilled water and antigen retrieval was performed. After cooling, the slides were placed in PBS (pH 7.4) and washed on a decolorizing shaker three times for 5 min each. After briefly air-drying the slides, the tissue outline was marked with a pen, and slides were blocked for 30 min with BSA. Slides were incubated overnight at 4°C with primary

antibody. On the following day, the slides were washed with PBS (pH 7.4) and incubated with the appropriate secondary antibody in the dark for 50 min. After washing slides three times for 5 min each with PBS (pH 7.4), DAPI staining solution (Servicebio, China) was applied. After washing the slides three times with PBS (pH 7.4), slides were incubated with fluorescence quenching agent B for 5 min. Slides were rinsed with running water for 10 min and sealed with anti-fluorescence quenching sealing agent. Slides were observed under a fluorescence microscope (Nikon, Japan). Photos were taken and analyzed using ImageJ software. Immunofluorescence staining was performed on sections prepared as described in Section 2.10, and results were examined under a fluorescence microscope and analyzed using ImageJ software.

1.8 Jess Capillary-Based Electrophoresis Immunoblot Assays

Jess Capillary-Based Electrophoresis Immunoblot Assays detailed steps: 1) Sample preparation: Lung tissue was lysed in RIPA buffer without SDS and supplemented with phosphatase and protease inhibitors. The tissue was homogenized and the lysate was kept on ice for 30 min, followed by centrifugation at 12,000 × g for 20 min at 4 °C. The supernatant was collected and protein concentration was determined using the BCA assay. A calculated volume of each sample was mixed with Loading Buffer, Reductant, and Sample Buffer from the Jess anti-rabbit detection module and diluted to a uniform concentration. The final volume of each diluted sample exceeded 4.5 μL. 2) Detection reagent preparation: The primary antibody was diluted 1:50 using the detection module diluent and kept on ice for subsequent use. 3) Microplate loading: The microplate was taken from the separation module. According to the manufacturer’s instructions, 3 μL of denatured sample was loaded into each capillary lane, followed by sequential addition of 5 μL of the provided marker, 10 μL of diluted primary antibody per sample, the supplied blocking solution, the detection module-provided secondary antibody, and 10 μL of Streptavidin-HRP or NIR. Finally, the total protein detection reagent was prepared, and 8 μL was added to each corresponding capillary lane. After loading all capillary lanes, wash buffer, chemiluminescent substrate, and the provided elution buffer (Replex) were added to the plate reservoirs. 5) Instrument run: The microplate was inserted into the

instrument, and the corresponding preset separation and detection method was selected in Compass software. The system automatically performed capillary electrophoresis, in-situ transfer, immunodetection, and chemiluminescent signal acquisition. 6) Data processing and normalization: Analysis was performed after verifying the fluorescent reference alignment. The software automatically integrated the specific peak area of each target protein and the full-spectrum total protein signal. Normalized relative expression was obtained by calculating the ratio of target protein signal to the corresponding total protein signal. The entire procedure was performed at an ambient temperature of 18–24 °C.

1.19. Enzyme Linked Immunosorbent Assay (ELISA)

ELISA detailed steps: 1) Preparation and equilibration: The kit was removed from 2–8°C and allowed to equilibrate at room temperature for 10 min. The required number of pre-coated strips was removed, and the remaining strips were sealed and stored at 4°C; 2) Sample addition and incubation: 100 μL of sample or standard of appropriate concentration was added to the assigned wells, and 100 μL of universal diluent was added to blank wells. The plate was sealed and incubated at 37°C for 60 min; 3) Detection antibody addition: The liquid in each well was discarded, and 100 μL of biotinylated detection antibody working solution was added directly to each well. The plate was sealed and incubated at 37°C for 60 min; 4) Washing: The liquid was discarded, 300 μL of 1× wash buffer was added to each well, allowed to stand for 1 min, then discarded. The plate was blotted dry on absorbent paper. This wash step was repeated three times; 5) Enzyme conjugate addition: 100 μL of streptavidin-HRP working solution was added to each well. The plate was sealed and incubated at 37°C for 30 min; 6) Washing again: The washing procedure described in step 4 was repeated five times; 7) Color development: 90 μL of TMB substrate was added to each well. The plate was sealed and incubated at 37°C in the dark for 15 min; 8) Termination and detection: 50 μL of stop solution was added to each well, and the absorbance of each well was measured immediately at 450 nm using a microplate reader (SpectraMax M5, Molecular Devices, USA).
